# The texture of narrative dilemmas: qualitative study in front-line professionals working with asylum seekers in the UK

**DOI:** 10.1192/bjb.2020.33

**Published:** 2021-02

**Authors:** Paaras Abbas, Martha von Werthern, Cornelius Katona, Francesca Brady, Yeree Woo

**Affiliations:** 1 Helen Bamber Foundation, UK; 2 Goldsmiths University of London, UK; 3 University College London, UK; 4 Central and North West London NHS Foundation Trust, UK

**Keywords:** Trauma, post-traumatic stress disorder, qualitative research, psychiatry and law, human rights

## Abstract

**Aims and method:**

Asylum seekers are required to narrate past experiences to the UK Home Office, doctors, lawyers and psychologists as part of their claims for international protection. The Home Office often cites perceived inconsistencies in asylum interviews as grounds for refusal of their claims. A number of processes affect asylum seekers' abilities to narrate past experiences fully to the professionals interviewing them. The dilemmas around disclosure that asylum seekers face have received little attention to date. This work aims to explore the perspectives of UK-based medico-legal report-writing doctors, lawyers and psychologists whose work involves eliciting narratives from asylum seekers on the processes that affect asylum seekers' abilities to disclose sensitive personal information in interview settings. Eighteen professionals participated in semi-structured interviews in individual or focus group settings to discuss, from their perspectives of extensive collective professional experience, the narrative dilemmas experienced by asylum seekers with whom they have worked.

**Results:**

Professionals identified a number of processes that made disclosure of personal information difficult for asylum seekers. These included asylum seekers' lack of trust towards the professionals conducting the interview, unclear ideas around pertinence of information for interviewers, feelings of fear, shame and guilt related to suspicions around collusions between UK and their country-of-origin's authorities, sexual trauma and, occasionally, their own involvement or collusion in crimes against others.

**Clinical implications:**

Recommendations are made on how to improve the interview environment to encourage disclosure. These have important implications for future research and policy initiatives.

The UK, as a signatory to the 1951 United Nations Convention and Protocol Relating to the Status of Refugees, has an obligation to consider applications from individuals fleeing persecution in their countries of origin.^1^ The UK Home Office assesses applications through initial and substantive interviews with asylum seekers, who are expected to narrate their past experiences to demonstrate their reasons for seeking asylum. The United Nations High Commissioner for Refugees defines an asylum seeker as ‘someone whose request for sanctuary has yet to be processed’.^2^ A refugee is defined as ‘someone who is unable or unwilling to return to their country of origin owing to a well-founded fear of being persecuted for reasons of race, religion, nationality, membership of a particular social group or political opinion’ according to the 1951 Refugee Convention.^1^ In the year ending June 2018, the UK Home Office received 27 044 applications for international protection and granted 14 308 of them.^3^ The Home Office sometimes rejects asylum claims based on perceived inconsistencies in asylum seekers' accounts of their past experiences.^4^ Asylum seekers may have to narrate these experiences to other professionals, including asylum tribunal judges (when rejected asylum claims are subject to appeal), medico-legal report-writing doctors and psychologists (for forensic reports to provide medical evidence in regards to the person's asylum claim), legal professionals (for witness statements), and psychologists and other therapists (for the treatment of mental health problems).

Asylum seekers' abilities to disclose information about their past experiences may be affected by various psychological and cultural factors, particularly where there is a history of trauma. Studies on resettled refugees indicate that asylum seekers who have sustained multiple traumas in the past often report high levels of post-traumatic stress disorder (PTSD) and depression.^5,6^ It is well documented that asylum seekers who have had many traumatic experiences will avoid disclosing such memories.^7^ Research suggests that external factors such as the interview room setting and the behaviour of Home Office officials can serve as triggers for traumatic memories.^8^ Currently, little is known about the range of dilemmas faced by asylum seekers when making decisions about the content of their narrated accounts, such as omitting or altering information, sometimes on the instructions of or under the influence of other people.^9^ Professionals working with asylum seekers offer a unique vantage point to guide an exploratory study of these dilemmas, as they are able refer to their extensive professional experience with a variety of asylum seekers. A small sample of individuals with refugee status were interviewed separately about dilemmas they experienced when narrating their past experiences to professionals. This constitutes a complementary study which is currently being prepared for submission.

## Method

### Participants

Eighteen professionals were recruited from fourteen different organisations that provide clinical and legal support to asylum seekers in England. The research study was advertised through direct email outreach requesting interviews with individuals whose work involves eliciting accounts from asylum seekers. Organisations were identified using chain sampling based on existing professional connections. Current and former Home Office decision makers were also invited to participate, but no individuals accepted the opportunity to do so.

Written informed consent was obtained from all participants. All procedures contributing to this work comply with the ethical standards of the relevant national and institutional committees on human experimentation and with the Helsinki Declaration of 1975, as revised in 2008. All procedures were approved by the University College London ethics committee (reference 8133/003).

### Data collection

Data were collected between July and December 2017 through face-to-face interviews, conducted either individually or as focus group interviews. Three professionally homogenous groups of doctors or lawyers were interviewed using a focus group discussion format. Table 1 shows the distribution of professionals. Focus group discussions were chosen as an appropriate method of data collection for a feasibility study because of their inherent ‘dynamic quality of group interaction’.^10^ Three therapists were interviewed individually owing to practical constraints.

**Table 1:** Characteristics of study participants

Participant	Research group	Occupation	Gender	Years in profession
P1	Therapist	Psychotherapeutic worker	Male	10
P2	MLR focus group 1	Psychiatrist – MLR writer for NGO	Male	37
P3	MLR focus group 1	Medical doctor – MLR writer for two NGOs	Male	22
P4	MLR focus group 1	Medical doctor - MLR writer for two NGOs	Female	57
P5	MLR focus group 1	General practitioner and MLR writer	Female	20
P6	Therapist	Psychologist at an NHS service and MLR writing volunteer for an NGO	Female	5
P7	Lawyer focus group	Legal officer for NGO	Male	30
P8	Lawyer focus group	Immigration solicitor at law firm	Male	13.5
P9	Lawyer focus group	Immigration solicitor at law firm	Female	11
P10	Lawyer focus group	Legal officer for international NGO	Male	Undisclosed
P11	Lawyer focus group	Immigration caseworker for law firm	Male	10
P12	Lawyer focus group	Immigration caseworker for law firm	Female	4+
P13	MLR focus group 2	Former general practitioner and MLR writer	Female	27
P14	MLR focus group 2	Clinical psychologist	Female	13
P15	MLR focus group 2	Director of NGO and clinical psychologist	Female	32
P16	MLR focus group 2	Former general practitioner, independent forensic physician	Female	30
P17	MLR focus group 2	Clinical psychologist	Female	12
P18	Therapist	Psychotherapist and director of NGO	Male	25

MLR, medico-legal report; NGO, non-governmental organisation; NHS, National Health Service.

Semi-structured interviews involving open-ended questions were conducted. The authors of this paper designed the interview schedule collaboratively, with input from a small team of psychologists and lawyers with critical insights into the effects of credibility concerns inherent to the UK's asylum adjudication on asylum narratives. The interviews were structured to encourage professionals to reflect on how asylum seekers they worked with experienced the process of narrating past experiences.

Questions included the following.

(a) Have you experienced situations where your clients' narratives evolved or changed over time?
(b) In what ways have these narratives changed?
(c) What do you think is the impact of the location in which asylum seekers are talking to professionals such as yourselves, and/or the presence of an interpreter, on the type of narrative that they are able to share?
(d) Are there any causes that you might be able to attribute to late disclosure of additional or changed information, based on your experiences?
(e) Do you think it's difficult for all asylum seekers to talk about what's happened to them in their past, or more for certain asylum seekers compared to others?

### Data analysis

Interviews were recorded and transcribed verbatim. A thematic analysis approach was used to identify patterns of experiences of different professionals and across the interviews.^11^ The data-set was double-coded by one author (P.A.) who collected the data and another (M.V.W.) who was not familiar with the content of the interviews prior to coding. The entire data-set was coded descriptively, and key themes were identified that were evident across the data-set. Two research questions drove decisions about the ‘keyness’ of a theme.^11^

The authors collaboratively interpreted codes and themes in the data. A third author (Y.W.) input the data into NVivo version 10. The research team were cognisant of the theoretical framing that drove the interview schedules and their own analytical skillset that informed the coding. Following Braun and Clarke's reflexive thematic analysis approach, the research team focused on ‘reflexive and thoughtful engagement with their data’.^12^

(a) What are the dilemmas faced by asylum seekers when narrating their past experiences to different professionals, as understood by professionals who work with them?
(b) What factors may inhibit an open discussion of the past experiences that asylum seekers are required to narrate in their claim?

The richness of the data is demonstrated in the Results section through the use of quotes from the data-set that illustrate the key themes in the data.

## Results

The four main themes of processes influencing asylum seekers' narratives that emerged from the interviews are reported here. These are:

(a) omission of information as a narrative dilemma;
(b) alteration of information as a narrative dilemma;
(c) embellishment of information as a narrative dilemma;
(d) influence of trauma on disclosure.

The term ‘narrative dilemmas’ refers to the dilemmas experienced by asylum seekers when narrating their past experiences to professionals interviewed for this research. Other issues that emerged from the data included the effects of individual identity and interview environment on disclosure. These are addressed in the Discussion section.

### Omission of information as a narrative dilemma

The majority of participants revealed that they had worked with asylum seekers who had omitted a piece of information at least once during their claim for asylum. A medico-legal report-writing doctor presented the example of an asylum seeker who had been imprisoned in a police station and jail, and refused to divulge details of her experiences in the police station. In his opinion:

‘This wasn't that she didn't remember. She clearly did. It was much more of a conscious choice of choosing not to re-engage with something that she knew she would find distressing. And there was a sort of moral component to it that she felt she didn't want to be defined by what had happened’ (P2, psychiatrist, male).

Participants described situations in which asylum seekers they worked with did not volunteer information. These included when the information was of a sensitive nature such as sexual trauma, if they were unclear about the pertinence of a piece of information for the interviewer, or when they were asked a direct question in one interview but not in another.

Participants expressed the collective view that asylum seekers who had perpetrated acts of betrayal or violence often did not disclose these experiences in the first interview or meeting with a professional. This was also the case for experiences of sexual violence (particularly for men). Participants attributed this to feelings of guilt, shame and stigma, as well as lack of trust in the professional. Participants reflected that that asylum seekers sometimes omitted information owing to fear of repercussions from people who exercised control over them, such as human traffickers or state authorities in their countries of origin whom they sometimes suspected of colluding with UK state authorities. According to participants, asylum seekers they worked with may doubt the confidential nature of interviews with UK professionals and as a result omit information.

### Alteration of information as a narrative dilemma

Participants shared a common experience that the asylum narratives they heard from their clients were altered over time. This often included delayed disclosure of some aspects of asylum seekers' past experiences. Information disclosed later would replace information that had been omitted as mentioned above or fabricated for reasons discussed below. Participants were of the opinion that asylum seekers sometimes disclosed more new information in the setting of an increasingly trusting relationship with the interviewer over time, on advice of a friend or family member, in response to changing levels of stigma in the community or in situations of external pressure such as being under oath in court.

Participants shared a variety of changed narratives that they had observed in their professional experience, from minor details such as changes of dates to the disclosure of new significant traumatic events such as rape. Examples reported included asylum seekers who said they escaped their country of origin in a specific month and then later changed it to another because they could recall the season but not the dates. In addition, they recounted incidents where some asylum seekers claimed that they entered the country later than they had, based on advice from friends to conceal the number of years spent undocumented before claiming asylum for fear that their claims would be refused on that basis.

A doctor interviewed an asylum seeker who, according to previous interview records, had allegedly been trafficked directly to the UK. However, she later disclosed that she had, in fact, been trafficked to a different country and had subsequently escaped and paid a smuggler to enter the UK. She had fabricated that part of her claim based on the advice of a friend.

A lawyer reported that he had seen many asylum seekers who had downplayed their membership of anti-government groups, based on unfounded warnings from others that asylum claims from members of such proscribed organisations were likely to be refused.

A male asylum seeker who had divulged information about a rape to the doctor preparing his medico-legal report called the doctor years later to have his account of this significant event redacted. He denied having ever mentioned it and wanted his statement altered, despite the doctor's records clearly documenting his previous account of the rape. The doctor attributed this to a wish to take control of the recollection of a past experience in which control had been taken away from him.

It was the common experience of participants that survivors of human trafficking had been given information by their traffickers, such as names of locations they had crossed in order to reach the UK, and that they had believed this information and repeated it in their interviews. Some survivors of trafficking had been specifically instructed to tell a fabricated story and complied because of threats, or owing to the power they perceived their trafficker to have over them. For example, several participants gave examples of traffickers who subjected individuals to ‘juju’ rituals exploiting their spiritual beliefs,^13^ in order that the individual would feel ‘bound’ to the trafficker and compelled to comply with their demands for fear of (often life-threatening) reprisals against themselves or loved ones.

‘I certainly have seen a number of victims of trafficking who are told that if they don't cooperate – for example, if they don't cooperate with the full story, which the trafficker has given them – then the trafficker will do the same thing to their younger sister as they did to them’ (P2, psychiatrist, male).

A medico-legal report-writing doctor had worked with an asylum seeker who drastically changed her narratives. He thought this was because the asylum seeker's abuser was present at the first interview, had acted as an interpreter for her and had controlled what was told to the doctor.

A further medico-legal report-writing doctor who visited asylum seekers in detention recalled receiving a phone call from an asylum seeker the day after the examination. The doctor reported that this asylum seeker had shared her decision to disclose new information that she had previously withheld about the sexual violence she had endured, after seeking the advice of a friend. Participant 6 explained:

‘With the minor details or changes in dates, and things, I would say that's memory. With more significant, kind of, omissions, I guess, I would say it's normally trust.’ (P6, psychologist, female)

Participants were of the opinion that sometimes the asylum seekers they worked with lied to protect the lives of others. Others who identified with their abusers may have wanted to protect the abusers and change their narratives accordingly.

### Embellishment of information as a narrative dilemma

A medico-legal report-writing doctor had seen a survivor of a ‘blood feud’ who had embellished his past experiences by fabricating that he was an only child. Whereas in the survivor's view this ‘would make my story stronger’, according to the doctor, it had the opposite effect when it was uncovered. Similarly, a lawyer shared his experience of what he viewed to be a recent trend:

‘We see a lot of these [nationality redacted] boys, they come from a background of very limited education, shepherds, and then have an experience of living in a conflict zone, domestic violence, their father's been murdered in front of them, and then maybe they've decided to add on a torture story because somebody's told them that won't get you asylum and you need to have been tortured [to get asylum]. Maybe they have been and maybe they haven't, but a whole lot of other bad stuff has happened to them.’ (P7, lawyer, male)

Psychologist participants explained that often when asylum seekers embellished their symptoms, there were likely clinical explanations for their behaviour, for example, the individual attempting to get help for their unmet needs.

‘The things she was presenting with were not consistent with any formal diagnosis … however, she desperately wanted to get her needs met but it was almost as though she'd heard other people had done it so therefore she put that into the pot to be sure that I would recognize that she needed help. That's how I interpreted it, actually’ (P13, doctor, female).

### Influence of trauma on disclosure

Participants reported that in some situations they interpreted the silence of an asylum seeker as indicative of the most severe parts of their traumatic history. Participants thought this inability to verbalise such experiences highlighted the effects of sustained periods of intimidation and subordination on survivors of trafficking and/or servitude and torture, such as the inability to develop an identity or narrative of their own. Sexual trauma, linked to feelings of embarrassment, shame and humiliation, was cited as one of the most difficult experiences for asylum seekers to disclose. Participants attributed this to fears of social stigma and resultant social exclusion. Participants reflected on their interactions with asylum seekers who they thought did not self-identify as victims of abuse, and expressed the opinion that the skewed self-perception of some asylum seekers affected how they presented certain experiences to their interviewers. This was particularly the case with victims of childhood abuse, according to participants, who may not have understood their experiences as constituting abuse and had fragmented memories of their past. Childhood trauma was explained as having particularly prolonged and longstanding effects on individuals, influencing their ability to disclose such events, and seen as potentially leading to intensified symptoms of PTSD. Re-experiencing phenomena and avoidant behaviours are core symptoms of PTSD.^14^ All participants spoke about the ‘fragmentation’ of memories that can occur in PTSD, which can lead to ‘gaps’ in the individual's narrated history, which in turn can lead to ‘inconsistent’ accounts.^15^

Participants across all interviews also spoke about their assessments of the effects of dissociation on the asylum seekers' ability to disclose a full and complete history. Some shared experiences where individuals had appeared to them to lose awareness of their surroundings and their sense of self, inevitably resulting in a lack of clarity or coherence in the narrative. Participants also analysed individuals' desire to avoid thinking about the traumatic memories as manifesting itself by avoiding talking about the traumatic event, and avoiding external reminders of the traumatic event, which complicated disclosure. For example, a participant shared her experience that clients often use vague expressions and euphemisms when talking about traumatic experiences instead of clearly disclosing a description of the sexual experiences.

‘People use vague terms as part of their PTSD defence and avoidance, about “them” or “that man”, “those people” or “the work”’ (P15, psychologist, female).

All professionals detailed a myriad of body language indicators of distress, including restlessness, reddening eyes, crying, movement of jaw muscles, hyperventilation and body clutching, as well as other PTSD symptoms described above. Participants continually underlined the importance of non-verbal cues in their professional roles in order to provide corroborating evidence for any clinical conclusions and noted that non-audible features of clients' accounts were mostly absent from transcripts of Home Office interviews.

The focus group discussions produced consensus over the majority of issues reported this section. There was a minor point of contention in the second focus group discussion with medico-legal report writing doctors regarding the balance between their roles as impartial witnesses and their urge to express their sympathy for the asylum seekers they work with.

## Discussion

This exploratory research investigated the processes involved in asylum narratives that changed over time from the perspectives of professionals who work with them. This study revealed the unique vantage points of doctors, lawyers and psychologists with years of collective experience working with asylum seekers from different countries with different types of asylum claims, in professional relationships ranging from singular interviews to long-term therapeutic work.

One of the key findings of this research is that, according to participants in this study, asylum seekers may sometimes select what information to disclose based on their perceptions of its pertinence to the particular interview or interviewer. This is particularly significant given that asylum seekers are interviewed by a range of different professionals and suggests that they make decisions about what information to share with each professional. Most participants conveyed that the time available to build a rapport with the asylum seekers they interviewed was directly related to the level of disclosure they subsequently received from the asylum seekers, owing to the development of a trusting relationship. The different professional standpoints and the difference in time available to participants, such as the length of the interview, the number of appointments, and the duration of the professional relationship, affected the type of information disclosed to them. The findings suggest that any expectation that asylum seekers make full disclosures of their past experiences is unrealistic because disclosure is a prolonged process which often cannot be achieved in a singular interview. Similarly, expectations around the accuracy of detail in a narrative, or consistency over time, are incompatible with the processes of human memory, particularly in individuals suffering from PTSD. Participants mentioned additional factors that contributed to narrative dilemmas experienced by the asylum seekers they worked with, which were in line with previous studies on the influence of the interview environment, including physical features of the room, the gender of the interviewer and the role of interpreters, as well as interviewer expectations of emotional congruence from asylum seekers.^9,16–21^ However, as our study focused on professionals' perceptions of the decision-making processes that drove some asylum seekers' disclosures, we chose to focus on types of dilemmas and their relationship with asylum seekers' traumatic past experiences.

This is a small sample study, whose results cannot be generalised. Furthermore, interviews did not follow an identical format, owing to adjustments made to accommodate individual participants' time constraints, which may have affected the content of the interviews. As this study adopted Braun and Clarke's reflexive thematic analysis method,^12^ a collaborative approach was taken that focused away from coding consistency and towards a nuanced reading of interview data.

A limitation of this study was that majority of the participants were white British; future research in this area should endeavour to recruit a more diverse sample, which would additionally allow another layer of analysis, i.e. of the relationship between professionals' ethnicity and asylum seekers' disclosures.

Overall, our findings demonstrate that it is potentially unrealistic to expect that asylum seekers will be able to disclose their past experiences in a complete manner to professionals, particularly in a single interview. It also highlights that there are a number of dilemmas that can affect asylum seekers' decisions around disclosure to professionals interviewing them, which are closely connected to their past traumatic experiences, present strong emotions about stigma and repercussions, and ongoing difficulties navigating the asylum process in the UK.

The scarcity of clear and accessible information about the procedures of asylum adjudication in the UK^11^ renders asylum seekers susceptible to unhelpful advice from well-meaning friends or instructions from controllers, as conveyed by participants who reported incidents where asylum seekers had altered or fabricated elements of their narratives as a result of advice from friends or instructions from controllers. This demonstrates the need to interrogate what appears to be a ‘choice’ on the part of the asylum seeker to disclose or not disclose information, when in fact this may be a distressing dilemma arising out of trauma, fear and the control exercised by others such as traffickers, or due to misinformation or misunderstanding about what is ‘best’ for their asylum case.

Participants considered that the experiences of asylum seekers who had been survivors of trafficking or modern slavery, sexual trauma, captivity in detention or prison, torture, and childhood traumas negatively affected their ability to discuss their past experiences openly. Such experiences were thought to render such individuals intimidated, devoid of power, mistrustful and suspicious. Our findings also suggest that details of traumatic events were sometimes not revealed to participants, and that on occasion traumatic events were revealed gradually over the course of a trusting professional relationship or following the perceived safety of successfully being granted leave to remain in the UK. These changes were seen by the professionals as a means by which asylum seekers exercised control over which aspects of their narrative they would share, and by doing so tried to regain some of the control that had been taken away from them when they had been subjected to abuse and torture in the past. Psychologists, psychiatrists and psychotherapeutic workers who had a professional understanding of the psychological needs of asylum seekers conveyed the importance of paying attention to the coherence between symptoms and the content of narratives to identify whether presentation of symptoms was a result of traumatic experiences or a misguided way of eliciting the help the individual felt they needed.

It is important to note that all participants spoke about the effects of fragmented trauma memories, which were said to lead to gaps in oral histories, leading to dislocated narratives and inconsistent accounts. These inconsistencies are sometimes cited as a reason for refusal in letters from the Home Office.^4^ Notably, a hostile asylum system and its ‘culture of disbelief’ was cited as a further source of trauma for some asylum seekers, who experienced anxieties as they anticipated being disbelieved or discredited; this supports recent literature on the effects of immigration detention.^22,23^

Our findings have a number of practical implications for professionals who interview asylum seekers, and for policy makers and researchers.

(a) The participants involved in this study conveyed that professionals conducting interviews can take measures to understand the process of disclosure in its complexity with the aim of making the interview environment conducive to gaining asylum seekers' trust and making them feel relaxed enough to disclose their full history. Participants recounted that certain individuals looked upon the notion of confidentiality with suspicion, or found it difficult to disclose sensitive personal information in front of professionals or interpreters from their own country and preferred instead to speak with foreign professionals. These are important factors to be considered when setting up an interview that requires disclosure of sensitive personal information. Participants' recommendations include considering in advance and where possible adjusting features of the interview setting to suit the specific needs of clients, such as lighting and noise levels, as well as the gender of the interviewer or interpreter; increasing interviewer awareness of PTSD symptomatology; and incorporating non-verbal information shared by the client, such as signs of distress or symptoms of PTSD, into interview notes.
(b) Participants conveyed that professionals working with asylum seekers should treat them as individuals rather than a category of people and engage with their individual needs and dilemmas in a non-judgemental manner. Participants in our study explained that they perceived it as their task to understand and clarify the content of narratives and the reasons for any inconsistencies, in order to place the narratives in the context of the individual's asylum claim. This is indicative of a broader structural issue relating to the asylum interview, which, by requiring an individual to produce a narrative that is continuously consistent, places more emphasis on synthesising often traumatic histories into a coherent format, rather than addressing the health and welfare needs relating to these traumas.
(c) We also recommend that future research focuses specifically on the experiences and opinions of asylum seekers and seeks their perspective on the issues that influenced any changes in their accounts over time.

## Data Availability

All authors had access to the study data (i.e. recordings and transcripts) for the purpose of analysis and interpretation. Access is ongoing until publication is completed. Anonymised data can be made available upon request from authors.

## About the authors

**Paaras Abbas** is a Research Administrator at the Department of Anthropology, Goldsmiths University of London and formerly a researcher at the Helen Bamber Foundation, London, UK. **Martha von Werthern** is a trainee clinical psychologist at the Department of Psychology, University College London and formerly a research coordinator at the Helen Bamber Foundation, London, UK. **Cornelius Katona** is Medical Director of the Helen Bamber Foundation, London and Honorary Professor of Psychiatry in the Division of Psychiatry, University College London, UK. **Francesca Brady** is a clinical psychologist at the Helen Bamber Foundation, London and at the Woodfield Trauma Service, Central and North West London NHS Foundation Trust, UK. **Yeree Woo** is a researcher at the Helen Bamber Foundation, London, UK.

## Supplementary material

For supplementary material accompanying this paper visit https://doi.org/10.1192/bjb.2020.33. click here to view supplementary material

## References

[1] UN General Assembly. Convention Relating to the Status of Refugees, Treaty Series, vol. 189: 137. United Nations. 1951. Available from: https://www.refworld.org/docid/3be01b964.html [accessed 17 Feb 2020].

[2] United Nations High Commissioner for Refugees. *Asylum-Seekers*. UNHCR. Available from: https://www.unhcr.org/uk/asylum-seekers.html.

[3] Home Office. *National Statistics: Summary of Latest Statistics*. Home Office, 2018. Available from: https://www.gov.uk/government/publications/immigration-statistics-year-ending-june-2018/summary-of-latest-statistics [accessed 9 Jul 2019].

[4] Trueman T. Reasons for refusal: an audit of 200 refusals of Ethiopian asylum-seekers in England. JIANL 2009; 23(3): 281–308.

[5] Fazel M, Wheeler J, Danesh J. Prevalence of serious mental disorder in 7000 refugees resettled in western countries: a systematic review. Lancet 2005; 365(9467): 1309–14. 15823380 10.1016/S0140-6736(05)61027-6

[6] Brewin CR, Dalgleish T, Joseph S. A dual representation theory of posttraumatic stress disorder. Psychol Rev 1996; 103(4): 670. 8888651 10.1037/0033-295x.103.4.670

[7] Bognor D. What prevents refugees and asylum seekers exposed to violence from disclosing trauma? Doctoral dissertation, University of London, 2005.

[8] Bögner D, Brewin C, Herlihy J. Refugees’ experiences of Home Office interviews: a qualitative study on the disclosure of sensitive personal information. J Ethnic Migr Stud 2010; 36(3): 519–35.

[9] Beneduce R. The moral economy of lying: subjectcraft, narrative capital, and uncertainty in the politics of asylum. Med Anthropol 2015; 34(6): 551–71. 26258605 10.1080/01459740.2015.1074576

[10] Smith JA, ed. Qualitative Psychology: A Practical Guide to Research Methods. Sage, 2015.

[11] Braun V, Clarke V. Using thematic analysis in psychology. Qual Res Psychol 2006; 3(2): 77–101.

[12] Braun V, Clarke V. Reflecting on reflexive thematic analysis. Qual Res Sport Exerc Health 2019; 11(4): 589–97.

[13] Office of the Special Representative and Co-ordinator for Combating Trafficking in Human Beings. *Trafficking in Human Beings Amounting to Torture and other Forms of Ill-treatment*. OSCE, 2013. Available from: https://www.osce.org/cthb/103085?download=true [accessed 17 Jul 2019].

[14] American Psychiatric Association. Diagnostic and Statistical Manual of Mental Disorders, Fifth Edition (DSM-5). APA, 2013.

[15] Brewin CR, Dalgleish T, Joseph S. A dual representation theory of posttraumatic stress disorder. Psychol Rev 1996; 103(4): 670. 8888651 10.1037/0033-295x.103.4.670

[16] Bögner D, Herlihy J, Brewin CR. Impact of sexual violence on disclosure during Home Office interviews. Br J Psychiatry 2007; 191(1): 75–81. 17602129 10.1192/bjp.bp.106.030262

[17] Herlihy J, Turner SW. The psychology of seeking protection. Int J Refugee Law 2009; 21(2): 171–92.

[18] Herlihy J, Jobson L, Turner S. Just tell us what happened to you: autobiographical memory and seeking asylum. Appl Cogn Psychol 2012; 26(5): 661–76.

[19] Bennett C, Thomas F. Seeking asylum in the UK: lesbian perspectives. Forced Migr Rev 2013; 42: 25–28.

[20] Rogers H, Fox S, Herlihy J. The importance of looking credible: the impact of the behavioural sequelae of post-traumatic stress disorder on the credibility of asylum seekers. Psychol Crime Law 2015; 21(2): 139–55.

[21] Gill N, Good A, ed. Asylum Determination in Europe: Ethnographic Perspectives. Springer, 2018.

[22] Robjant K, Hassan R, Katona C. Mental health implications of detaining asylum seekers: systematic review. Br J Psychiatry 2009; 194(4): 306–12. 19336779 10.1192/bjp.bp.108.053223

[23] von Werthern M, Robjant K, Chui Z, Schon R, Ottisova L, Mason C, The impact of immigration detention on mental health: a systematic review. BMC Psychiatry 2018; 18(1): 382. 30522460 10.1186/s12888-018-1945-y PMC6282296

